# Poisoning by *Nerium oleander* L. in Franconia Geese

**DOI:** 10.3390/ani14040612

**Published:** 2024-02-14

**Authors:** Nicola Pugliese, Antonella Tinelli, Giuseppe Crescenzo, Maria Nieddu, Elena Baralla, Antonella Schiavone, Nicola Zizzo, Rossella Samarelli, Filomena Dessì, Elena Circella, Claudia Zizzadoro, Medhat S. Saleh, Antonio Camarda

**Affiliations:** 1 Department of Veterinary Medicine, University of Bari, 70010 Valenzano, Italy; nicola.pugliese@uniba.it (N.P.); giuseppe.crescenzo@uniba.it (G.C.); antonella.schiavone@uniba.it (A.S.); nicola.zizzo@uniba.it (N.Z.); rossella.samarelli@uniba.it (R.S.); elena.circella@uniba.it (E.C.); claudia.zizzadoro@uniba.it (C.Z.); medhat.elshahat@uniba.it (M.S.S.); antonio.camarda@uniba.it (A.C.); 2 Department of Medicine, Surgery and Pharmacy, University of Sassari, 07100 Sassari, Italy; marvi@uniss.it; 3 Department of Veterinary Medicine, University of Sassari, 07100 Sassari, Italy; ebaralla@uniss.it (E.B.); f.dessi@studenti.uniss.it (F.D.); 4 Department of Animal Production, Faculty of Agriculture, Benha University, Benha 13736, Egypt

**Keywords:** Franconia goose, *Nerium oleander*, acute poisoning, oleandrin

## Abstract

**Simple Summary:**

This research investigates the poisoning of domestic geese by oleander plants, commonly used as ornamentals. Oleander is toxic and can be fatal to various animals, including geese. The study focuses on a case in which four geese were accidentally poisoned after ingesting oleander clippings. The affected geese exhibited sudden and severe symptoms, leading to death within 15–90 min. Necropsy and histopathology revealed damage to the heart and kidneys, while oleandrin, a toxic compound in oleander, was detected in the heart, kidney, brain liver, and muscles. The findings highlight the acute toxicity of oleander in geese and emphasize the need for awareness in goose-rearing practices to prevent exposure to potentially lethal plants. This information can guide measures to protect domestic birds and may have broader implications for animal husbandry practices, contributing to the well-being of domestic fowl and other susceptible animals.

**Abstract:**

This study describes the acute poisoning of four 3-month-old Franconia geese (*Anser anser*) by oleander plants (*Nerium oleander*). After the accidental ingestion of oleander clippings, the geese exhibited a rapid onset of severe symptoms, leading to mortality within 15–90 min. Necropsy revealed cardiac and renal lesions. Specifically, interstitial edema, red blood cell infiltration, and myofibril loss were observed in the cardiac muscle, and tubular epithelial degeneration, interstitial edema, and hemorrhages were evident in the kidneys. Oleandrin, a glycoside with cardiac effects, was detected in the liver, kidneys, heart, brain, and muscles. The clinical implications underscore the urgency of veterinary intervention upon oleander ingestion, and the specific findings contribute valuable insights into the pathological effects of acute oleander poisoning in geese, aiding veterinarians in prompt diagnosis and treatment.

## 1. Introduction

Oleander (*Nerium oleander* L. 1753) is an evergreen perennial plant belonging to the *Apocynaceae* family, which originated in the Mediterranean area and is currently cultivated as an ornamental plant worldwide [1]. *N. oleander* is toxic to both invertebrates and vertebrates, being one of the leading causes of animal or human poisoning by plants [2]. Its primary cytotoxic components are cardiac glycosides belonging to cardenolides, such as neriifolin, oleandrin, and digitoxigenin [3], whose levels are high in fresh or dried seeds, stems, twigs, leaves, roots, and root barks [4,5]. Glycosylated metabolites are very common among plants, and they have often constituted a starting point for the development of beneficial compounds [6]. A wide number of plant genera contain cardiac glycosides [7]. Their action is mainly exerted through the inhibition of Na^+^/K^+^ ATPase, which causes an increase in the sodium and, consequently, calcium concentrations in cardiomyocytes through the action of the sodium–calcium exchanger [8]. More recent studies have also hypothesized the involvement of stretch-activated Ca^2+^ permeable cation channels [9]. The increase in the intracellular Ca^2+^ concentration induces a positive inotropic effect [10]. Additionally, bradycardia, ventricular and supraventricular arrhythmia, and depression of the ST segment in electrocardiogram have been observed [3]. Furthermore, oleander poisoning leads to nausea, vomiting, abdominal pain, diarrhea, hyperkalemia, weakness, and drowsiness [11]. Toxic effects on the central nervous system (CNS) have also been reported [12].

Accidental and experimental cases of oleander poisoning have been described in several species, such as horses [13], chickens [14], cattle [15], dogs [16], and other livestock species [17]. A brief abstract also reported the poisoning of about 1000 geese with oleander, but without providing further clinical, pathological, or diagnostic information [18].

Considering the scarcity of information about the effects of oleander poisoning in avian species, this study aimed to report the pathological and toxicological features of *N. oleander* poisoning in domestic geese due to accidental ingestion.

## 2. Materials and Methods

### 2.1. Case Description

Nine 3-month-old Franconia geese (a light breed of the greylag goose *Anser anser*) were reared as game birds in a fenced area with two adults. The breeding area also hosted ornamental chickens (*Gallus gallus*) and pigeons (*Columba livia*), reared in separate pens. In total, about 30 birds were reared. The area where geese lived harbored several plants belonging to non-toxic species (i.e., *Prunus amygdalus*, *Juglans regia*, *Laurus nobilis*, *Ficus carica*, and *Schinus molle*). A long hedge along the borders of the enclosure consisted of *N. oleander*, while the lawn was covered by *Cynodon dactylon*.

The day the poisoning occurred, the oleander hedge was pruned, together with the other plants, and clippings were not removed immediately but roughly chopped and left on the ground to be hauled the next day.

In the late afternoon, four out of the nine 3-month-old Franconia geese (ring codes 169, 170, 171, and 176) suddenly crouched and began hyperventilating and shaking their head. Two of them (ring codes 169 and 170) also exhibited neurological signs, which consisted of slow and wide oscillations of the head. Mild sialorrhea was observed in geese 170 and 171.

A few minutes after the first signs, the affected geese tried to rise by violently flapping their wings. When they reached the erect posture, their locomotion was weaving, with hyperextension of the neck. After taking a few steps, animals fell and died within seconds (Supplementary Material). The entire sequela took about 10–15 min for geese 169, 170, and 176, and about 90 min for the fourth (ring code 171). The remaining animals, namely the other five young geese, the adults, and the other reared birds, showed no signs of intoxication.

Following the suggestion of the avian pathologists, the owner removed the oleander clippings and no other cases occurred.

### 2.2. Necropsy and Histopathology

The carcasses were brought to the Department of Veterinary Medicine of the University of Bari, where a necropsy was performed three hours after the death of the animals.

For histopathology, tissue samples were collected and fixed in 10% neutral buffered formalin, routinely processed, and embedded in paraffin wax using an automatic tissue processor (Reichert-Jung Gmbh, Nussloch, Germany). Serial 5 µm sections from all samples were cut with a 2030 Biocut microtome (Reichert-Jung, Nussloch, Germany), mounted on Super Frost glass slides (Menzel-Glaser, Braunschweig, Germany), and then stained with standard hematoxylin and eosin (HE) (Bio-Optica, Milan, Italy). All sections were examined using a light microscope (Leika DM4000B, Milan, Italy) equipped with a digital C-mount camera TP1080HDWI (Alexasoft, Florence, Italy).

### 2.3. Oleandrin Detection and Quantification

To confirm the oleander intoxication and provide toxicological information, oleandrin was detected and quantified. Oleandrin was chosen because, among the cardenolides produced by *Nerium oleander*, it is the most known, the most studied, and the most abundant. Additionally, oleandrin is the substance that is usually detected and quantified in oleander poisoning cases [19,20,21]. Samples of heart, liver, kidney, brain gastric muscle, and thigh muscle were collected and immediately stored at −20 °C for oleandrin detection and quantification. The analysis was carried out from the samples of geese 169, 170, and 176, while samples from goose 171 were lost.

Stock standards solutions (1 mg/mL) of oleandrin (Merck, Milan, Italy) and the internal standard (IS) testosterone-D3 (Merck) were prepared in methanol and stored at +4 °C. All proper dilutions were made in the mobile phase, consisting of 1:1 acetonitrile (Merck) and ultrapure water. The method was linear in the range of 10–100 ng/mL (y = −0.000813 + 0.016321x; R^2^ = 0.9999). The IS concentration was 20 ng/mL, and the limit of detection was 5 ng/mL.

Extraction was performed from all the tissue samples as previously described [15] with modifications. Briefly, 0.5 g wet weight of homogenized goose tissues was transferred into a 50 mL tube with 5 mL of acetonitrile. Samples were vortexed for 30 min, put in an ultrasonic bath for 30 min, and then centrifuged at 1400× *g* for 8 min. The supernatant was collected in a clean tube and dried under a nitrogen stream. The residue was reconstituted with 1 mL of the IS solution in the mobile phase and injected into the LC-MS apparatus. Each sample was extracted twice and analyzed in duplicate.

Chromatographic analyses were performed using a UPLC Dionex Ultimate 3000Q coupled with an Exactive Orbitrap mass spectrometer (ThermoScientific, Milan, Italy).

The chromatographic process was carried out using a Poroshell 120 EC-C18 column (3.0 × 100 mm, 2.7 μm, Agilent, Santa Clara, CA, USA). The mobile phase consisted of MilliQ water with 0.1% formic acid (solvent A) and acetonitrile (solvent B). A linear gradient was performed with a flow of 0.350 mL/min, as follows: after 1 min at 5%, solvent B was increased to 100% in 4 min, and maintained at a constant rate for 3 min; in 0.1 min, solvent B was decreased to 5% and kept at this percentage to re-equilibrate the system for 2 min. The injection volume was set at 5 μL.

The retention time of oleandrin was 4.8 min. Analytes were detected with heated electrospray ionization (HESI-II) in positive mode. The optimized HESI-II temperature was set at 320 °C, and the capillary temperature at 300 °C. The electrospray voltage was set at 10 kV. Sheath and auxiliary gases were 35 and 15 arbitrary units, respectively. The acquisition was achieved in full scan/dd-MS2. All quantitative data were calculated using the full scan data. The mass range in the full scan was within m/z 250–1000. The data were acquired at a resolution of 70,000 full widths at half maximum (FWHM) (m/z 200). The mass spectrometer was controlled by Xcalibur 3.0 software (ThermoScientific). The concentrations of the extracts were measured as ng/mL and converted into ng of oleandrin per g of wet matrix. They were expressed in the text as the mean of the repetitions ± standard deviation. One-way ANOVA and Tukey HSD post hoc tests were applied to verify if there were significant differences among the oleandrin concentrations in the organs of each goose.

### 2.4. Bacteriological and Virological Investigations

To exclude avian influenza or Newcastle disease, well known for causing acute disease and sudden mortality, distinct RT-PCRs were performed targeting the matrix gene of the influenza A virus and the fusion protein gene of the avian avulavirus serotype 1, respectively, as previously described [22,23]. The template consisted of total RNA extracted from each tissue sample by the RNeasy mini kit (Qiagen, Milan, Italy) according to the manufacturer’s instructions. Similarly, a loopful of liver, kidney, spleen, and lung were separately streaked on blood agar plates to exclude potential bacterial systemic infections. Plates were incubated at 37 ± 1 °C for 24 h.

## 3. Results

All RT-PCRs for the detection of influenza A virus and avian avulavirus were negative, and no bacterial growth was observed on blood agar plates.

**Figure 1:**
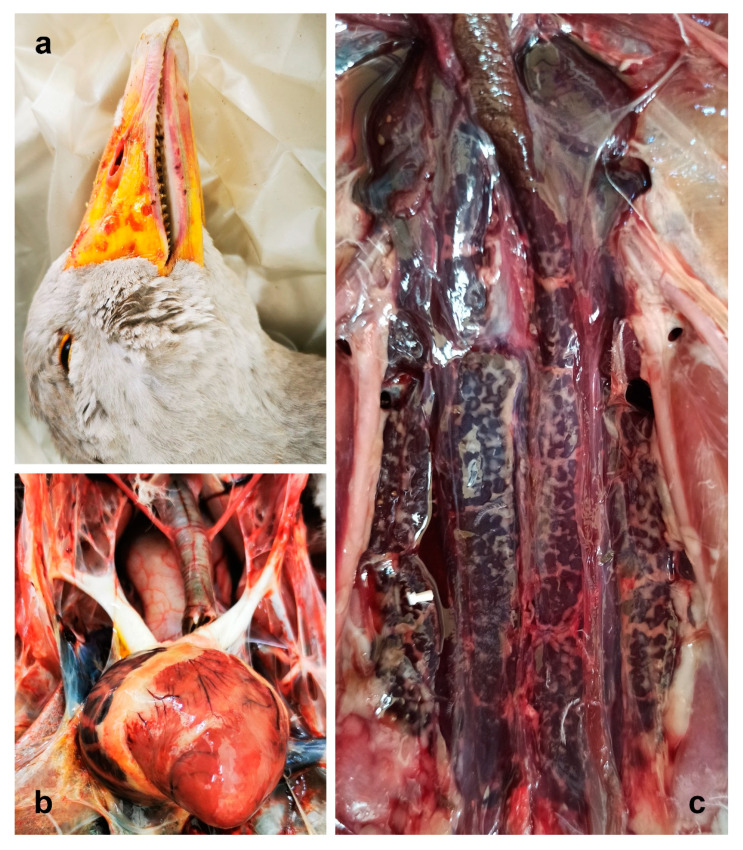
Gross lesions. (**a**) Beak of goose 170: petechial hemorrhages are evident (**b**) Heart of goose 171, mottled with hemorrhagic areas. (**c**) Kidney of goose 176: swelling and dark red discoloration can be observed.

At necropsy, all dead geese appeared normal in feathering and well fleshed (Supplementary Figure S1a), with a thick layer of adipose tissue and optimal development of the pectoral muscles. Petechial hemorrhages were observed on the beak of geese 170, 171, and 176 (Figure 1a), and hyperemic spots were visible on the upper proximal region of the beak in one goose (169), along with sialorrhea signs. A scrap of plant material apparently belonging to an oleander leaf lay on the palate and tongue of the latter goose. (Supplementary Figure S1b).

When exposed, the organs in the visceral cavity did not show evident alteration, except for the goose 171, which presented with general congestion, with the heart being mottled, with greyish and white foci and hemorrhagic areas. The right atrium was congested, globose, and enlarged (Figure 1b).

Tracheas and primary bronchi were pervious in all animals and no inflammatory signs were observed. Likewise, air sacs were unaltered in all subjects, and visceral serosa was regular and not thickened. The livers of geese 169 and 176 were enlarged with round edges, and pale focal areas were evident. The liver of goose 171 was congested, other than enlarged, while no evident gross lesions were observed in the liver of goose 170. Conversely, the kidneys of all geese were congested, bilaterally swollen, and discolored with dark red (Figure 1c). Spleen, pancreas, intestines, reproductive organs, and Bursa of Fabricius presented no remarkable alterations.

**Figure 2:**
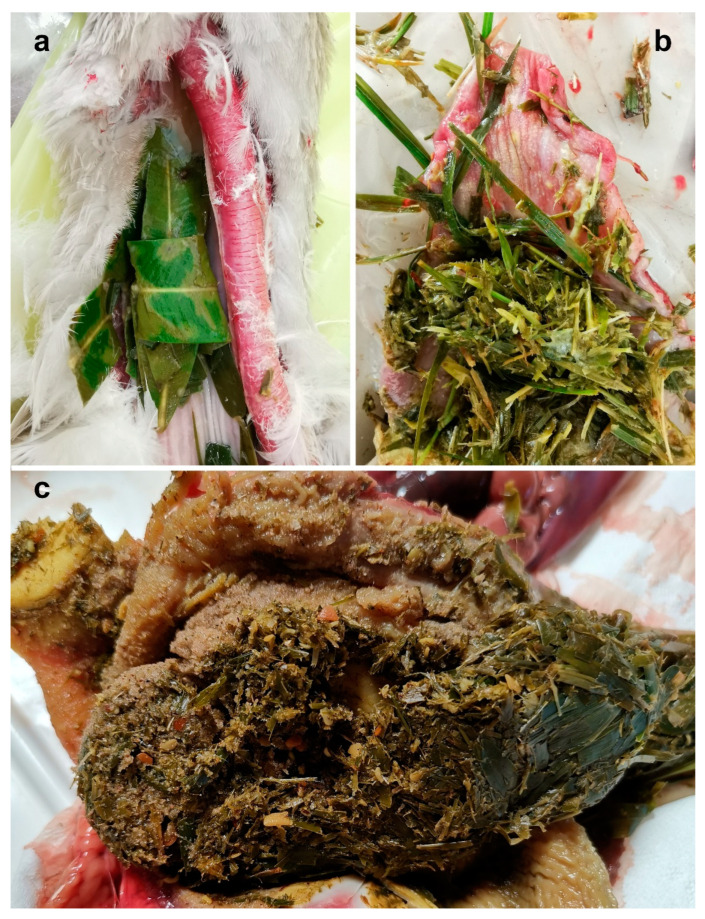
Fragments of oleander leaves mixed with other vegetal material in the higher esophagus of goose 176 (**a**), proventriculus of goose 171 (**b**), and gizzard of goose 176 (**c**).

The esophagus of all animals presented slight hyperemia. Roughly chopped oleander and *C. dactylon* leaves were found in the lumen of geese 169, 170, and 176, along with residues of other plants (Figure 2a), probably mixed with oleander during the pruning procedures.

No plant material was found in the esophagus of the goose 171, but more finely chopped material, resembling oleander leaves, was found in the proventriculus (Figure 2b).

A bolus of partially digested material and remains of leaves filled the gizzard of all animals (Figure 2c). The gastric muscle was not visibly altered.

**Figure 3:**
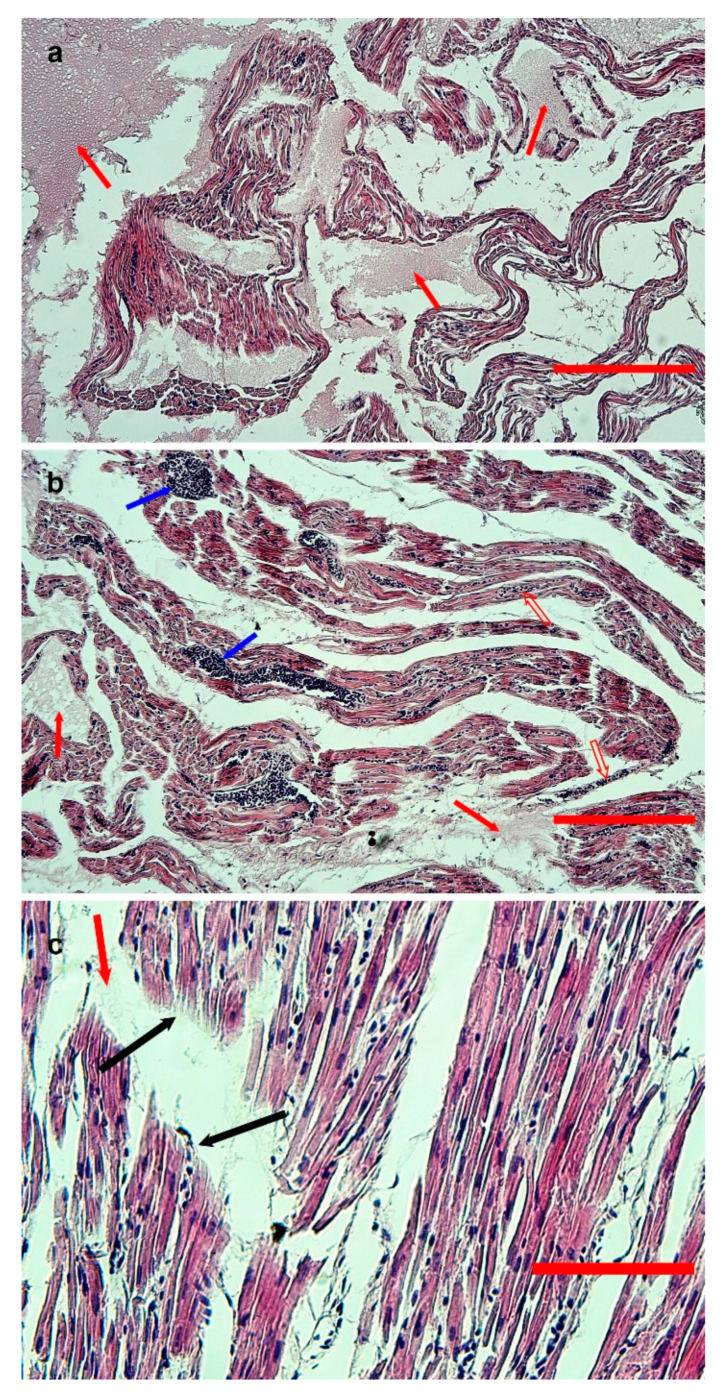
Hematoxylin-eosin-stained sections from formalin-fixed, paraffin-embedded samples of the heart of goose 170. (**a**,**b**) Low-magnification images (bar = 300 and 200 μm, respectively). Severe edema (red arrows) and moderate red blood cell infiltration (empty red arrows) are evident. Vessels are congested (blue arrows). (**c**) High-magnification image (bar = 80 μm), with evidence of rupture of myofibrils (black arrows) and edematous areas (red arrow).

On microscopic examination, the cardiac muscle showed interstitial edema, moderate red blood cell infiltration (Figure 3a,b), and loss of continuity of myofibrils (Figure 3c); pyknotic myocytes were also visible in the cardiac tissue of goose 171.

**Figure 4:**
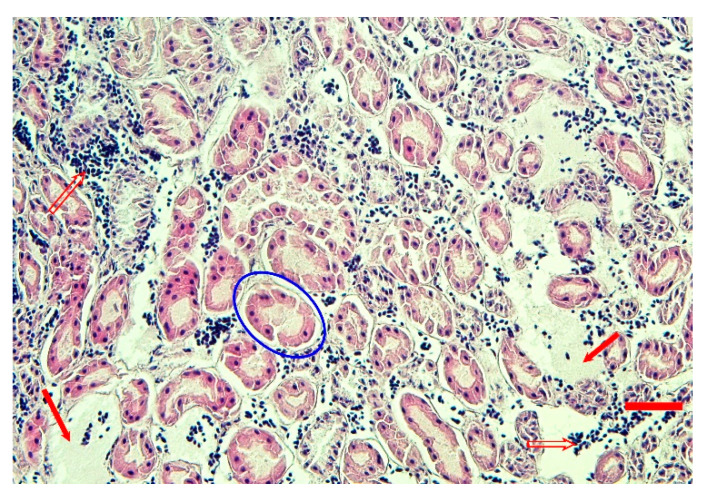
Hematoxylin-eosin-stained sections from formalin-fixed, paraffin-embedded sample of the goose 171 kidney. Interstitial edema (red arrows), hemorrhage (empty red arrows), and mild tubular degeneration (blue oval) are evident (bar = 40 μm).

Congestion of the kidneys was also evident at the microscopic level in all four geese; mild tubular epithelial degeneration, interstitial edema, and hemorrhages were observed (Figure 4).

No significant microscopic lesions were observed in the specimens from the other organs, including brain, of all four animals.

Oleandrin was detected in all the tested organs (Table 1 and Supplementary Table S1), including brains, at a concentration ranging from 32.7 ± 10.3 ng/g (about 53.49 ± 16.84 nmol/kg of tissue) in the gastric muscles of the goose 176 to 1130.2 ± 41.6 ng/g (about 1846.78 ± 68.0 5 nmol/kg) in the kidney of goose 170.

**Figure 5:**
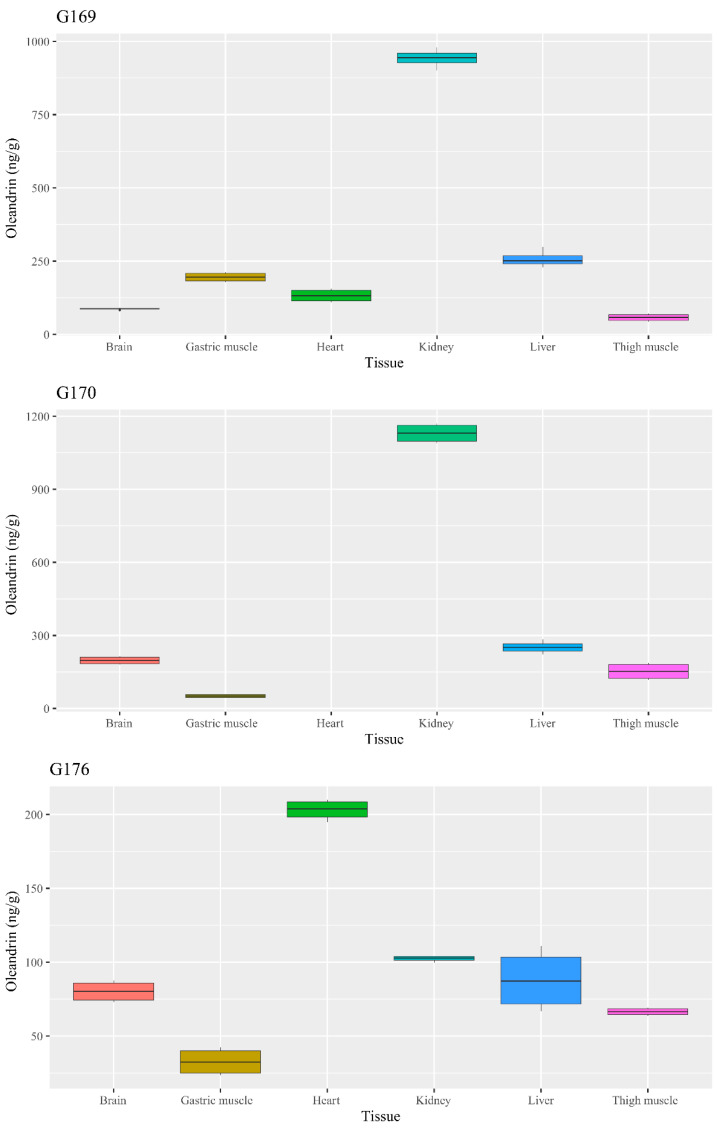
Boxplot chart of the oleandrin concentration in the analyzed geese tissues.

In two cases, goose 170 and goose 176 (Figure 5), the oleandrin level was significantly higher in the kidneys (*p* < 0.001). In goose 176, the highest concentration of oleandrin was recorded in the heart (*p* < 0.001), while the concentration in the kidney was comparable to that in the brain (*p* = 0.071) and liver (*p* = 0.413), and significantly higher than those in the gastric (*p* < 0.001) and thigh (*p* = 0.002) muscle.

## 4. Discussion

Altogether, all pieces of evidence strongly suggested acute oleander poisoning, which was consistent with the event history, the signs, the observed lesions, and the oleandrin level detected in the geese’s organs.

Cardiac lesions were evident at necropsy only in one goose (171), but they were histologically observed not only in that bird but also in the other three. This finding is consistent with a recent case report, which found no evident cardiac sign but an increase in biochemical markers of cardiac lesions, namely CPK, AST, LDH, and troponin I [24]. Additionally, the microscopic findings at the cardiac level agreed with the previous observations in broiler chickens [14] and in goats [25], which evidenced necrosis of cardiomyocytes with the presence of pycnotic nuclei and rupture of myofibrils. It is interesting to underline that the animals used in previous experimental intoxication died several hours after the oleander administration while, in the case presented here, animals died within 15–90 min. The absence of gross cardiac lesions might be due to the very short time between the toxin ingestion and the death of the animals, which prevented the development of more evident tissue damage.

Similarly, the renal lesions observed in the experimental poisoning of chickens were more severe than those described here. Omidi and colleagues [14] recorded severe tubular necrosis, hemorrhage, and congestion, while geese kidneys exhibited mild tubular degeneration with interstitial edema and hemorrhage. They could be due to blood circulation defects related to heart fibrillation, but a direct effect of oleandrin cannot be excluded. In fact, previous reports associated the leakage of lysosomal enzymes with deleterious effects on glomeruli and renal tubules in rats [26]. The high concentration of oleandrin measured from kidneys in geese supports this hypothesis, and this may be a distinctive point, as previous studies suggested a minor accumulation of oleandrin in kidneys of mice [27]. Such an apparent discrepancy might be due to different, species-specific pharmacokinetic properties of oleandrin in birds. Indeed, data about the oleandrin concentration in birds, and, more generally, in animals, are scarce. Oleandrin concentration was found to be about 15 ng/g in the heart muscle and liver of intoxicated cows [28], while, in a human fatal case, 10–30 μg/g of oleandrin was found in tissues after autopsy [29], consistent with later findings, which evidenced that micromolar concentrations of oleandrin and its metabolite, oleandrigenin were needed to exert their lethal effects [30].

On the other side, the histologic lesions in the heart and kidneys may be related to the functional inhibition, typical of the oleander toxins, of the Na^+^/K^+^ ATPase, which alters, in turn, the Ca^2+^ intracellular balance [31]. This osmotic impairment may consequently modify the cell volume, thus causing cellular damage via shrinkage and swelling. The blockade of the Na^+^/K^+^ ATPase induces, at the cardiac level, electrolytic alterations that compromise signal conduction [32]. The 50% inhibitory concentration of oleandrin and oleandrigenin on porcine Na^+^/K^+^ ATPase was measured in vivo at 0.62 and 1.23 μmol/L, respectively [30]; therefore, a greater susceptibility of geese to oleandrin might be hypothesized, since a lower concentration was detected in all organs except the kidneys.

The potential cardiac effects of the oleander toxins, which are well known for causing sinus bradycardia, inhibition of the atrioventricular node, and ventricular fibrillation [11], may also explain the signs observed in the goose 171, which died 90 min after the onset of the clinical signs.

The rapid occurrence of death probably did not allow for the development of further anatomic pathologic signs, except for some changes observed in the liver. Previous reports described hepatic lesions in intoxicated chickens, suggesting a possible necrotic effect of the oleander toxins [14]; in that case, animals died after 3–24 h, providing enough time to produce more severe damage. This was confirmed by the observation of additional congestion in goose 171, which survived about 90 min after the onset of signs. Finally, the observed esophagus hyperemia could be due to the stasis of plant material, which might have caused mechanical lesions.

Despite the clear indications of acute and hyperacute poisoning, doubts remain about the reasons for the timing of the clinical development. Alfonso and colleagues [18] reported that oleander poisoning led to death in geese in about 30 min, but individual variations are possible. In particular, it should whether this could be due to the high quantity of oleander ingested by the geese, or to intrinsic variations in the susceptibility of Franconia goose to the oleander toxins, should be verified. The literature data do not seem to suggest an increased susceptibility of birds. The lethal dose for mammals ranges from 50–100 mg/kg for horses to 200–220 mg/kg for ovine, with higher variability observed in dogs (50–220 mg/kg) and bovine (50–150 mg/kg) [33]. A review of the available data [34] highlighted that a lethal dose of oleander might range from 120 to 700 mg for different avian species, but the lack of kinetic data does not allow for a more accurate estimation of oleandrin lethality.

## 5. Conclusions

The present case provides useful information regarding the anatomopathological and toxicological aspects of oleander toxicosis in geese. Considering all the above information, it is possible to hypothesize that some avian species, such as *A. anser,* may be highly susceptible to the toxic action of oleandrin. This should be taken into account when organizing settlements for goose-rearing to avoid exposing geese to oleander or other plants producing potentially lethal cardiac glycosides.

## Figures and Tables

**Table 1:** Mean oleandrin concentration ± standard deviation detected in the geese tissues.

	Oleandrin Concentration (ng/g of Tissue)		
	Goose 169	Goose 170	Goose 176
Gastric muscle	195.2 ± 16.7	50.9 ± 7.7	32.7 ± 10.3
Thigh muscle	57.6 ± 13.2	151.9 ± 35.6	66.5 ± 2.6
Brain	87.4 ± 3.1	197.8 ± 17.2	80.2 ± 7.3
Liver	257.6 ± 29.9	252.1 ± 26.2	88.1 ± 21.3
Heart	132.5 ± 23.1	NA	203.1 ± 7.1
Kidney	942.6 ± 33.3	1130.2 ± 41.6	102.4 ± 2.1

NA: Not available.

## Data Availability

The original contributions presented in the study are included in the article and in the Supplementary Material; further inquiries can be directed to the corresponding author.

## Supplementary Materials

The following are available online at https://www.mdpi.com/article/10.3390/ani14040612/s1, Supplementary Video S1: Video showing the clinical signs exhibited by one of the geese involved in the poisoning event. Supplementary Figure S1: External appearance of the birds. (a) Goose 176 with well-developed pectoral muscle areas and flourishing plumage. (b) Scraps of oleander leaves on the palate and the tongue of the goose 169. Supplementary Table S1: Raw UPLC data of oleandrin concentration in the geese’s tissues.
