# Supplementary figures and images for: Poisoning by Nerium oleander L. in Franconia Geese
Source: Animals (Basel). 2024 Feb 14;14(4):612. doi: 10.3390/ani14040612 (PMC10885877; doi:10.3390/ani14040612)

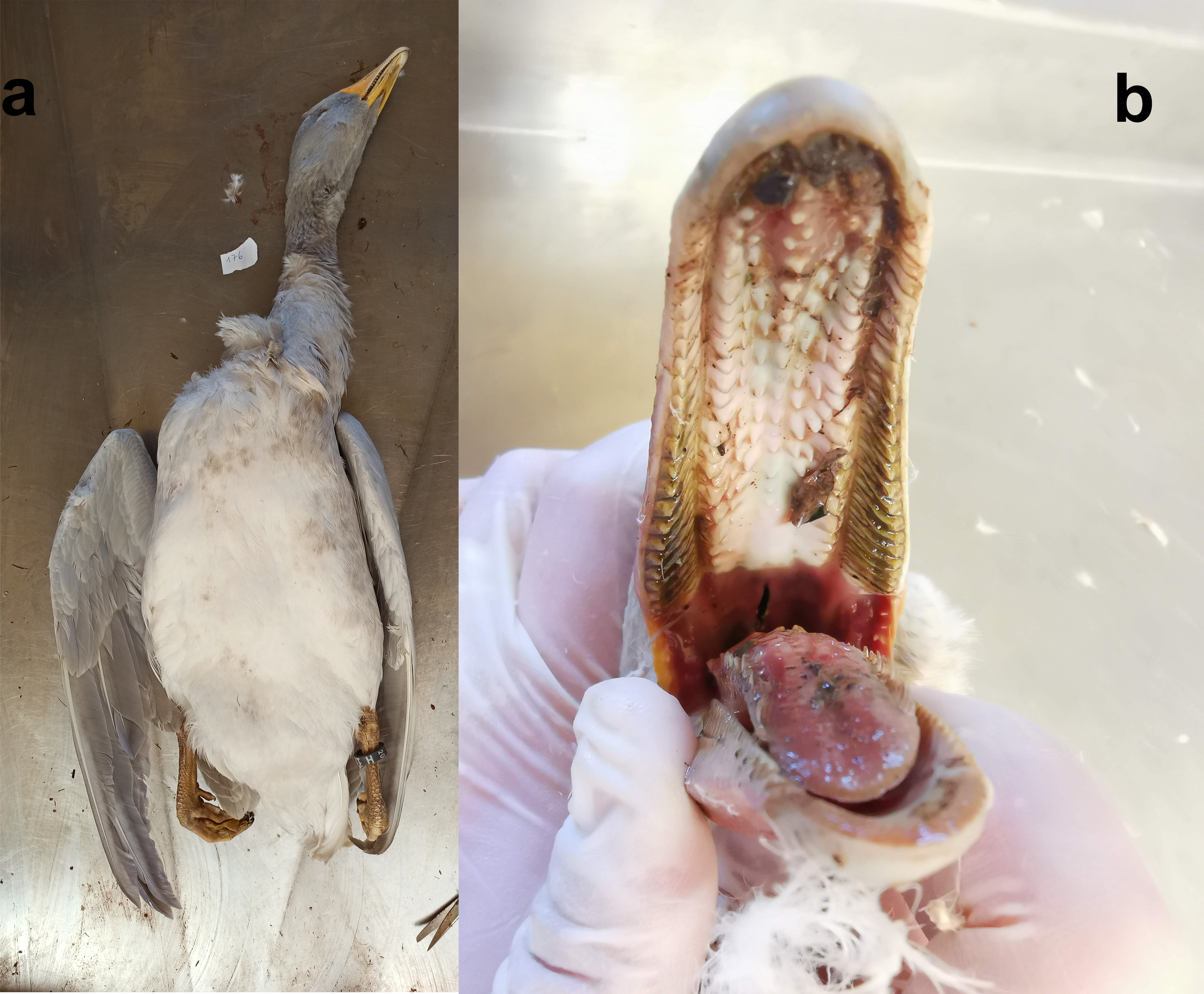

Supplement: Supplementary file 1 [file animals-14-00612-s001.zip › FigureS1.tif]
